# Association of non-contrast CT markers with long-term functional outcome in deep intracerebral hemorrhage

**DOI:** 10.3389/fneur.2023.1268839

**Published:** 2024-01-11

**Authors:** Kathrin Kölbl, Stefan W. Hock, Mingming Xu, Jochen A. Sembill, Anne Mrochen, Stefanie Balk, Stefan Lang, Bastian Volbers, Tobias Engelhorn, Bernd Kallmünzer, Joji B. Kuramatsu

**Affiliations:** ^1^Department of Neurology, University Hospital Erlangen, Friedrich-Alexander-Universität Erlangen-Nürnberg (FAU), Erlangen, Germany; ^2^Department of Neuroradiology, University Hospital Erlangen, Friedrich-Alexander-Universität Erlangen-Nürnberg (FAU), Erlangen, Germany

**Keywords:** intracerebral hemorrhage, functional outcome, hematoma expansion, imaging biomarker, non-contrast CT

## Abstract

**Objective:**

Hematoma expansion (HE) is the most important therapeutic target during acute care of patients with intracerebral hemorrhage (ICH). Imaging biomarkers such as non-contrast CT (NCCT) markers have been associated with increasing risk for HE. The aim of the present study was to evaluate the influence of NCCT markers with functional long-term outcome and with HE in patients with deep (basal ganglia and thalamus) ICH who represent an important subgroup of patients at the highest risk for functional deterioration with HE due to the eloquence of the affected brain region.

**Methods:**

From our prospective institutional database, all patients maximally treated with deep ICH were included and retrospectively analyzed. NCCT markers were recorded at diagnostic imaging, ICH volume characteristics were volumetrically evaluated, and all patients received follow-up imaging within 0–48 h. We explored associations of NCCT makers with unfavorable functional outcome, defined as modified Rankin scale 4–6, after 12 months and with HE. Bias and confounding were addressed by multivariable regression modeling.

**Results:**

In 322 patients with deep ICH, NCCT markers were distributed as follows: irregular shape: 69.6%, heterogenous density: 55.9%, hypodensities: 52.5%, island sign: 19.3%, black hole sign: 11.5%, and blend sign: 4.7%. Upon multivariable regression analyses, independent associations were documented with the functional outcome for irregular shape (aOR: 2.73, 95%CI: 1.42–5.22, *p* = 0.002), heterogenous density (aOR: 2.62, 95%CI: 1.40–4.90, *p* = 0.003) and island sign (aOR: 2.54, 95%CI: 1.05–6.14, *p* = 0.038), and with HE for heterogenous density (aOR: 5.01, 95%CI: 1.93–13.05, *p* = 0.001) and hypodensities (aOR: 3.75, 95%CI: 1.63–8.62, *p* = 0.002).

**Conclusion:**

NCCT markers are frequent in deep ICH patients and provide important clinical implications. Specifically, markers defined by diverging intra-hematomal densities provided associations with a 5-times higher risk for HE and a 2.5-times higher likelihood for unfavorable functional long-term outcome. Hence, these markers allow the identification of patients with deep ICH at high risk for clinical deterioration due to HE.

## Introduction

Spontaneous intracerebral hemorrhage (ICH) is one of the most severe subtypes of stroke (1–3). Currently, treatment options remain limited, as available randomized clinical trials have shown no effective treatment strategies (4). The most important possibly modifiable outcome predictor is hematoma expansion (HE) (5–7). In deep ICH (basal ganglia and thalamus) particularly, HE may be even more important, a specific subgroup with a poorer prognosis *per se*, due to higher reported HE rates linked to more frequent intraventricular hemorrhage (IVH) and its anatomical location involving more eloquent structures as compared to most lobar ICH regions (8–11). Specifically, according to recent studies, the odds for poor functional outcome and extent of disability-adjusted life-years were higher in deep ICH with HE as compared to lobar ICH patients with HE (12, 13).

Increasingly, imaging parameters are applied to select patients at the highest risk for HE, who may represent ideal candidates for therapeutic interventions; e.g., data from the Antihypertensive Treatment of Acute Cerebral Hemorrhage II (ATACH-2) trial suggested that patients with a high risk of HE benefit to a larger degree from intensive blood pressure reduction than those with low risk (14).

Recent studies have suggested that non-contrast CT markers are associated with HE as well as clinical outcomes and hence may represent a safer, faster, and valid alternative to approaches using CT contrast media (15, 16). The most commonly used NCCT markers include the following: density markers (heterogenous density, hypodensities, black hole sign, and blend sign) and shape markers (irregular shape and island sign) (17). According to a recent meta-analysis including 25 studies, statistical heterogeneity of these NCCT markers associated with investigated outcomes was moderate to substantial (HE: *I*^2^ span = 77–93% or functional short-term outcome: *I*^2^ span = 85–88%), with large variations in sample sizes and limited confounder adjustments in the individual studies (15).

Therefore, we decided to investigate associations of NCCT markers in a large cohort of patients with deep ICH (1) for functional long-term outcome at 12 months and (2) for HE on follow-up imaging, both after rigorous confounder adjustments. We analyzed a variety of density and shape NCCT markers based on data derived from a large prospective cohort study in ICH [NCT03183167].

## Methods

### Patient selection

**Figure 1:**
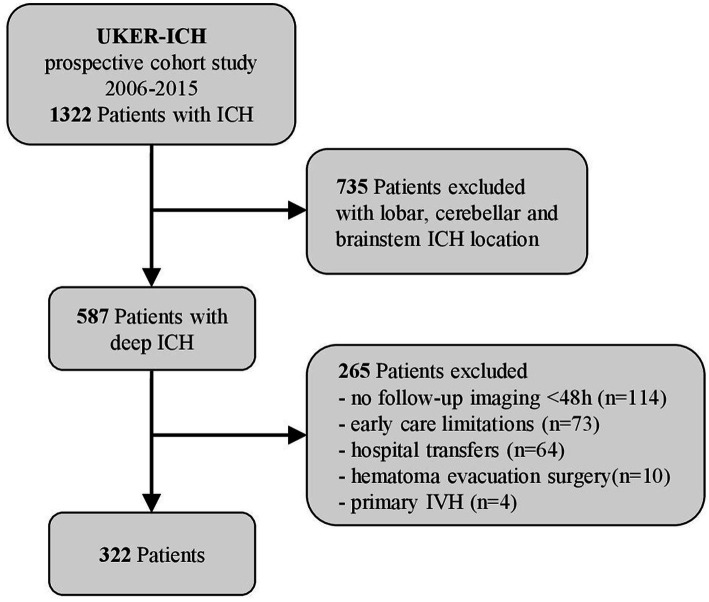
Flow chart of study participants. Overall, data of 1,322 patients with intracerebral hemorrhage (ICH) (2006–2015) were screened. After the exclusion of 735 patients with lobar, cerebellar and brainstem ICH, 587 patients with deep ICH (basal ganglia and thalamus) remained; 265 patients with no follow-up imaging <48 h (*n* = 114), early care limitations (*n* = 73), hospital transfers (*n* = 64), hematoma evacuation surgery (*n* = 10), and primary intraventricular hemorrhage (*n* = 4) were excluded. Therefore, 322 patients remained for data analyses.

Based on our prospective UKER-ICH registry (NCT03183167) in spontaneous ICH patients, we screened 1,322 patients with ICH treated at a single University Hospital between 2006 and 2015 (12, 18) and excluded 735 patients with infratentorial or lobar ICH location. To be able to analyze all patients who had full details of diagnostic (standardized DICOM formatted imaging) and follow-up imaging available (conducted within 0–48 h) for quantitative volumetric assessment and evaluation of NCCT markers, we further excluded 265 patients (*n* = 114) with missing follow-up imaging within 48 h, (*n* = 73) with established early care limitations (withhold, withdrawal of care within 24 h), (*n* = 64) who were transferred from outside hospitals, (*n* = 10) who received hematoma evacuation surgery, and (*n* = 4) with primary IVH. Hence, 322 solely deep ICH patients remained for this present analysis; please see Figure 1 flow diagram. The study was approved by the responsible ethics committee (115_17B), and informed consent was obtained from all individuals.

### Data collection

We retrieved data on demographics, baseline characteristics, and clinical parameters from our prospective study. Clinical data including age, gender, medical history [hypertension, congestive heart failure, previous brain infarction, previous bleeding complications, hepatic impairment, renal insufficiency, diabetes mellitus, and prior use of antiplatelet drugs (APT) and oral anticoagulant (OAC)], and status at hospital admission [pre-stroke mRS (pre-mRS), Glasgow Coma Scale score (GCS), the National Institutes of Health Stroke Scale Score (NIHSS), and systolic, diastolic, and mean (MAP) arterial blood pressure] were retrieved. The max-ICH Score was calculated as appropriate using clinical data and imaging (19).

### Imaging acquisition and analysis

Diagnostic and follow-up CT scans were performed according to institutional standardized protocols. DICOM data of all patients were retrospectively extracted and reanalyzed to evaluate NCCT markers and ICH volumes, measured by semi-automated planimetry as reported (20). Two experienced reviewers (SH, SL) who were blinded to all clinical information independently evaluated the presence of NCCT imaging markers. Discrepancies were settled by the third rater (TE). All NCCT signs were defined according to the diagnostic criteria described by Morotti et al. (17). Density markers such as heterogenous density (17, 21), hypodensities (17, 22), blend sign (17, 23), and black hole sign (17, 24), as well as shape markers such as irregular shape (17, 21) and island sign (17, 25) were assessed, as appropriate. Heterogenous density was defined as ≥3 hypoattenuated regions compared with the surrounding hematoma (using the categorial density scale from Barras). Two or more irregularities, joined or separated from the hematoma edge, were interpreted as irregular shape (using the categorial shape scale from Barras). Both markers were evaluated on the axial slice with the largest ICH area (17, 21). Any hypodense region with no connection to the outer surface of the hematoma, regardless of its size, morphology, or density, was characterized as hypodensity (17, 22). The island sign was defined as ≥3 scattered small hematomas, which are entirely separate from the main hematoma, or ≥ 4 small hematomas, some or all of which are connected to the main hematoma (17, 25). The blend sign was labeled as a relatively hypoattenuated region next to a hyperattenuated region within the hematoma with a well-defined margin and a density difference of at least 18 Hounsfield units (HU) (17, 23). The black hole sign was defined as a hypoattenuated area of any dimension or morphology that has a density difference of at least 28 HU with the encapsulating hematoma (17, 24, 26).

### Primary and secondary outcomes

We defined the primary outcome as the proportion of patients with unfavorable functional outcome at 12 months using the modified Rankin Scale (mRS [range: 0, no symptoms to 6, death]); unfavorable outcome was defined as a score of 4–6 (4, unable to walk unassisted, to 6, death), which was dichotomously compared with the proportion of patients with favorable outcome (0, no deficit, to 3, being able to walk unassisted) (27, 28). Follow-up information was gathered by personnel blinded to clinical data according to the study protocol (28). The secondary outcome consisted of the presence of HE. We defined hematoma expansion (HE) as a 6 mL absolute and/or 33% relative increase in hematoma volume measured at follow-up CT (0–48 h) (17).

### Statistical analysis

All statistical analyses were performed using STATA (Version 14.2).^1^ A value of *α = 0.05* was considered statistically significant. Categorial variables were presented as numbers and percentages (*n*, %) and were compared by Pearson χ^2^ and Fisher exact tests. The distribution of the data was evaluated by the Kolmogorov–Smirnov test: normally distributed continuous data [expressed as mean ± standard deviation (SD)] was compared using the Student *t*-test; otherwise, we used the Mann–Whitney U-test [presented as median and interquartile range (IQR)]. Sensitivity analyses to identify confounders with the investigated outcomes consisted of a dichotomized approach (mRS 0–3 vs. 4–6 and HE present vs. absent) to compare baseline characteristics. To evaluate independent associations of NCCT markers with primary and secondary outcomes, we conducted two multivariable binary logistic regression analyses (model A, mRS 4–6, model B, HE present). In general, confounders for each outcome were identified based on sensitivity analyses and evaluated by standardized mean difference >10% and according to existing evidence (5, 29–32). Hence, the following parameters were included in multivariate modeling: model (A): age, baseline ICH volume, pre-mRS, NIHSS, HE, and IVH; and model (B): baseline ICH volume, systolic blood pressure, APT, OAC, time from symptom onset to diagnostic CT, and time from diagnostic CT to follow-up CT. We graphically displayed these associations as Forest plots with corresponding adjusted odds ratios (aORs).

## Results

### Baseline characteristics

The entire study population consisted of 322 patients with deep ICH meeting selection criteria (Table 1), with a median age of 69 years (IQR, 59.6–77.0) and 40.1% (*n* = 129/322) being female. Prior use of antiplatelet drugs (APT) was found in 29.5% (*n* = 95/322), and prior use of oral anticoagulant (OAC) in 14.0% (*n* = 45/322). Neurological assessment on admission was measured by the NIHSS at a median of 15 (IQR, 9–24) and with a median max ICH score of 4 (IQR, 3–5). Diagnostic CT was performed at a median of 3.6 h (IQR, 1.6–8.5 h) after symptom onset, and follow-up CT was performed at 21.5 h (IQR, 14.2–28.4 h). Hematoma volume at diagnosis was at a median of 11.97 mL (IQR, 5.1–27.0 mL), IVH was found in 61.5% (*n* = 198/322) of patients, and HE occurred in 14.3% (*n* = 46/322). Unfavorable functional outcome at 12 months, defined as mRS 4–6, occurred in 61.8% (*n* = 199/322) of patients. For the sensitivity analysis of excluded patients, please see Supplementary Table S1.

**Table 1:** Baseline characteristics for the entire cohort and dichotomized according to long-term outcome (12 months).

	All (*n* = 322)	mRS 0–3 (*n* = 123)	mRS 4–6 (*n* = 199)	Absolute difference (95%CI)	SMD
Age, median (IQR)	69.0 (59.6–77.0)	65.0 (54.0–72.0)	72.0 (62.0–79.0)	7.0 (3.6 to 10.4)	0.60
pre-mRS, median (IQR)	1 (0–2)	0 (0–1)	1 (0–2)	1.0 (0.6 to 1.4)	0.57
Female sex, *n* (%)	129 (40.1%)	46 (37.4%)	83 (41.7%)	4.3 (6.6 to 15.3)	0.09
**Medical history, *n* (%)**					
Arterial hypertension	297 (92.2%)	115 (93.5%)	182 (91.5%)	−2.0 (−7.9 to 3.8)	−0.08
Heart failure	47 (14.6%)	14 (11.4%)	33 (16.6%)	5.2 (−2.4 to 12.8)	0.15
Prior ischemic stroke/TIA	67 (20.8%)	17 (13.8%)	50 (25.1%)	11.3 (2.7 to 19.9)	0.29
Prior ICH/bleeding complications	22 (6.8%)	6 (4.9%)	16 (8.0%)	3.2 (−2.2 to 8.5)	0.13
Hepatic insufficiency	31 (9.6%)	11 (8.9%)	20 (10.1%)	1.1 (−5.4 to 7.7)	0.04
Renal failure	52 (16.1%)	13 (10.6%)	39 (19.6%)	9.0 (1.3 to 16.8)	0.25
Diabetes mellitus	94 (29.2%)	38 (30.9%)	56 (28.1%)	−2.8 (−13.0 to 7.5)	−0.06
APT	95 (29.5%)	31 (25.2%)	64 (32.2%)	7.0 (−3.1 to 17.0)	0.15
OAC	45 (14.0%)	13 (10.6%)	32 (16.1%)	5.5 (−1.9 to 13.0)	0.16
**Status at hospital admission**					
GCS, median (IQR)	12 (6–14)	14 (11–15)	11 (4–13)	−3.0 (−4.8 to −1.2)	−0.72
NIHSS, median (IQR)	15 (9–24)	9 (5–16)	18 (12–32)	9.0 (5.9 to 12.1)	0.81
Syst. blood pressure, median (IQR)	170 (150–190)	171 (155–190)	170 (148–192)	−1.0 (−10.2 to 8.2)	−0.11
Dia. blood pressure, median (IQR)	92 (80–106)	95.0 (81.0–108.0)	90.0 (80.0–105.0)	−5.0 (−10.5 to 0.5)	−0.24
Mean blood pressure, mean (SD)	119.0 (22.5)	120.5 (21.2)	118.1 (23.3)	1 (−5.8 to 7.8)	−0.11
maxICH Score, median (IQR)	4 (3–5)	2 (1–4)	5 (4–6)	3.0 (2.6 to 3.4)	1.46
**Timing (hours), median (IQR)**					
Symptom onset to admission	3.2 (1.2–8.0)	3.3 (1.2–8.7)	3.2 (1.1–7.3)	−0.1 (−1.3 to 1.1)	−0.01
Symptom onset to diagnostic CT	3.6 (1.6–8.5)	3.6 (1.6–9.2)	3.6 (1.6–8.2)	0.0 (−1.2 to 1.2)	−0.01
Diagnostic CT to follow-up CT	21.5 (14.2–28.4)	22.3 (15.8–31.1)	20.9 (13.2–28.2)	−1.4 (−4.1 to 1.3)	−0.12
**Imagine**					
1. ICH volume (mL), median (IQR)	11.97 (5.1–27.0)	6.1 (2.0–14.9)	16.9 (8.4–35)	10.7 (6.6 to 14.9)	0.70
IVH, *n* (%)	198 (61.5%)	49 (39.8%)	149 (74.9%)	35.0 (24.5 to 45.6)	0.76
2. ICH volume (mL), median (IQR)	12.4 (5.0–32.5)	5.8 (2.1–15.3)	18.0 (8.0–44.2)	12.3 (6.9 to 17.7)	0.81
HE 33% and/or 6 mL, *n* (%)	46 (14.3%)	12 (9.8%)	34 (17.1%)	7.3 (−0.1 to 14.7)	0.22

Comparison of patients with favorable functional long-term outcome (mRS 0–3 score at 12 months) vs. patients with unfavorable functional long-term outcome (mRS score 4–6 at 12 months). Absolute differences are provided in percent for frequency data and for scales or continuous variables as mean differences of the according measurement unit. Standardized mean differences (SMD) are given to compare the difference between patients with favorable vs. poor outcomes. *n*, number of patients; SD, standard deviation; IQR, Interquartile range; CI, confidence interval; SMD, standardized mean differences; ICH, intracerebral hemorrhage, IVH, intraventricular hemorrhage; APT, prior antiplatelet drug use; OAC, prior oral anticoagulation drug use; premRS, modified Ranking scale sore before stroke; GCS, Glasgow Coma Scale (ranging from 3, comatose, to 15, alert); NIHSS, National Institutes of Health Stroke Scale (ranging from 0, no stroke symptoms, to 42, severe stroke); syst. Blood pressure, systolic blood pressure; dia. Blood pressure, diastolic blood pressure, mean arterial blood pressure, mean arterial blood pressure; max-ICH Score; maximally treated ICH Score; HE, Hematoma enlargement.

### Long-term outcome and NCCT markers

The dichotomized comparison (mRS 0–3 vs. 4–6) provided that patients with unfavorable outcome showed significant imbalances (Table 1), e.g., older age [72.0 (62.0–79.0) vs. 65.0 (54.0–72.0); absolute difference (AD): 7.0, 95%CI (3.6 to 10.4) years; standardized mean difference (SMD): 0.6], and worse pre-mRS [1 (0–2) vs. 0 (0–1); AD: 1.0, 95%CI (0.6 to 1.4); SMD: 0.57], more frequent prior ischemic stroke/TIA [25.1% (50/199) vs. 13.8% (17/123); AD: 11.3, 95%CI (2.7 to 19.9); SMD:0.29]. Patients with unfavorable long-term outcome had a worse neurological assessment on admission, i.e., GCS [11 (4–13) vs. 14 (11–15); AD: −3.0, 95%CI (−4.8 to −1.2); SMD: −0.72], NIHSS [18 (12–32) vs. 9 (5–16); AD: 9.0, 95%CI (5.9–12.1); SMD: 0.81], and max-ICH score [5 (4–6) vs. 2 (1–4); AD: 3.0, 95%CI (2.6–3.4); SMD: 1.46]. Patients with mRS 4–6 at 12 months had larger ICH volumes at diagnostic CT [16.9 (8.4–35) vs. 6.1 (2.0–14.9); AD: 10.7, 95%CI (6.6–14.9); SMD: 0.7] and follow-up CT [18.0 (8.0–44.2) vs. 5.8 (2.1–15.3); AD: 12.3, 95%CI (6.9–17.7); SMD: 0.81]. IVH and HE were found more often in patients with unfavorable outcome [IVH; 74.9% (149/199) vs. 39.8% (49/123); AD: 35.0, 95%CI (24.5–45.6); SMD: 0.76; and HE, 17.1% (34/199) vs. 9.8% (12/123); AD: 7.3, 95%CI (−0.1 to 14.7); SMD: 0.22].

The presence of the NCCT markers in all patients and dichotomized according to long-term outcome at 12 months (mRS 0–3 vs. mRS 4–6) is shown in Table 2. Any NCCT marker (presence of at least 1 NCCT marker) was present in 79.2% (*n* = 255/322) of patients. The frequencies of NCCT markers were as follows: 69.6% of all patients (*n* = 224/322) exhibited irregular shape, 55.9% (*n* = 180/322) heterogenous density, 52.5% (*n* = 169/322) hypodensities, 19.3% (*n* = 62/322) island sign, 4.7% (*n* = 15/322) blend sign, and 11.5% (*n* = 37/322) black hole sign. Patients with unfavorable long-term outcome showed more frequently NCCT markers than patients with favorable outcome [88.4% (176/199) vs. 64.2% (79/123); AD: 24.2, 95%CI (14.6 to 33.8); SMD: 0.59]. In patients with unfavorable outcome, the following NCCT markers were significantly more frequent: Irregular shape [80.4% (160/199) vs. 52.0% (64/123); AD: 28.4, 95%CI (18.0 to 38.8); SMD: 0.63], heterogenous density [66.8% (133/199) vs. 38.2% (47/123); AD: 28.6, 95%CI (17.8 to 39.4); SMD: 0.60], hypodensities [60.3% (120/199) vs. 39.8% (49/123); AD: 20.5, 95%CI (9.5 to 31.5); SMD: 0.42], island sign [26.1% (52/199) vs. 8.1% (10/123); AD: 18.0, 95%CI (10.2 to 25.8); SMD: 0.49].

**Table 2:** Distribution of NCCT markers in the entire cohort and dichotomized according to long-term outcome (12 months).

NCCT markers, *n* (%)	All (*n* = 322)	mRS 0–3 (*n* = 123)	mRS 4–6 (*n* = 199)	Absolute difference (95%CI)	SMD
Any sign	255 (79.2%)	79 (64.2%)	176 (88.4%)	24.2 (14.6 to 33.8)	0.59
Irregular shape	224 (69.6%)	64 (52.0%)	160 (80.4%)	28.4 (18.0 to 38.8)	0.63
Heterogenous density	180 (55.9%)	47 (38.2%)	133 (66.8%)	28.6 (17.8 to 39.4)	0.60
Hypodensities	169 (52.5%)	49 (39.8%)	120 (60.3%)	20.5 (9.5 to 31.5)	0.42
Island sign	62 (19.3%)	10 (8.1%)	52 (26.1%)	18.0 (10.2 to 25.8)	0.49
Blend sign	15 (4.7%)	4 (3.3%)	11 (5.5%)	2.3 (−2.2 to 6.7)	0.11
Black hole sign	37 (11.5%)	9 (7.3%)	28 (14.1%)	6.8 (0.1 to 13.4)	0.22

Distribution of NCCT (non-contrast CT) markers in the entire cohort and dichotomized according to long-term outcome at 12 months (mRS 0–3 vs. mRS 4–6). Absolute differences are provided in percent for frequency data and for scales or continuous variables as mean differences of the according measurement unit. Standardized mean differences (SMD) are given to compare the difference between patients with favorable vs. poor outcomes. *n*, number of patients; CI, confidence interval; SMD, standardized mean differences; NCCT, non-contrast CT; Any sign, the presence of at least 1 NCCT marker.

**Figure 2:**
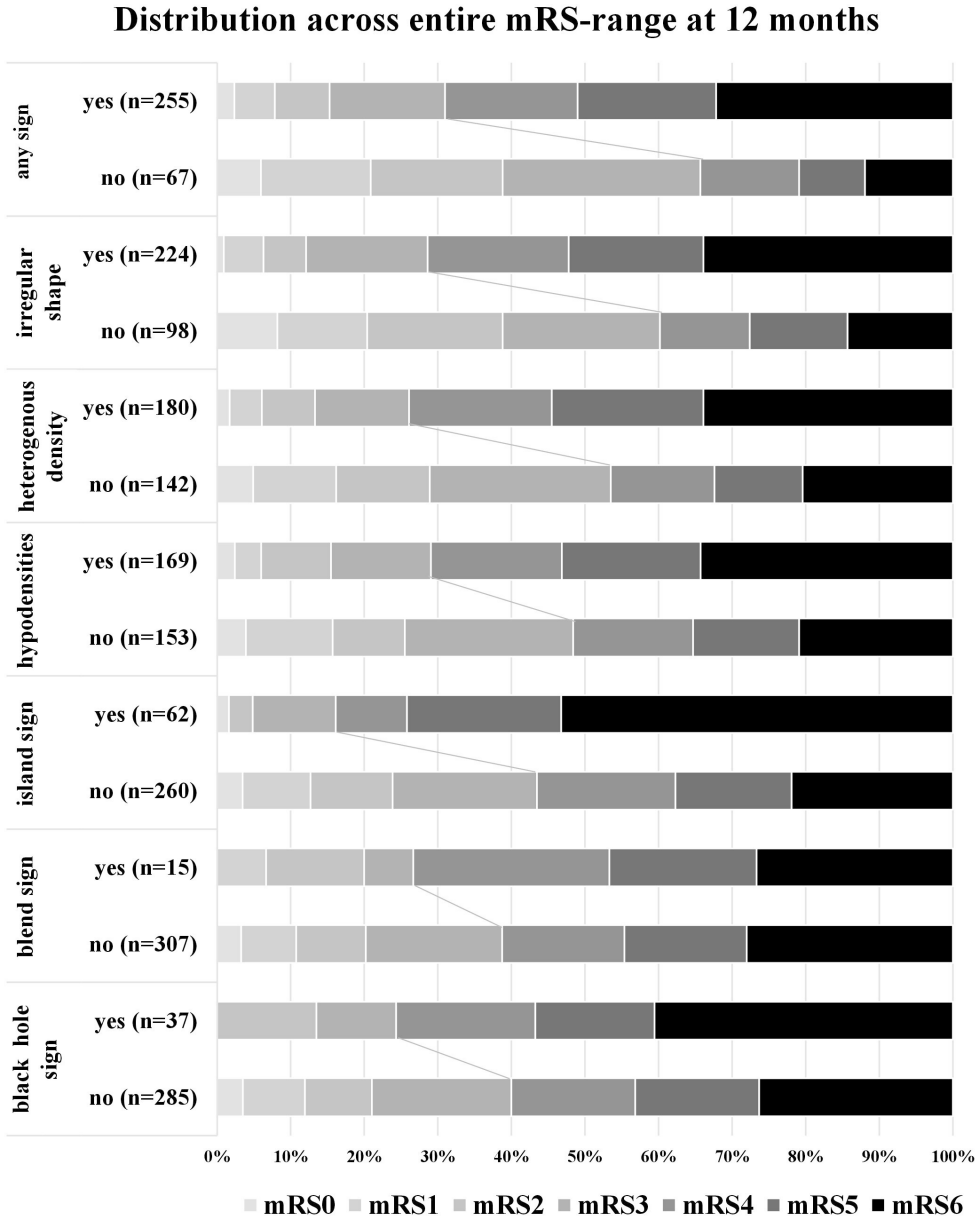
Distribution of functional long-term outcome at 12 months. Distribution of modified Rankin Scale (mRS) at 12 months in patients according to the presence of non-contrast CT (NCCT) markers. The percentage of participants with the mRS obtained at 12 months is shown in each cell. Any sign, the presence of at least 1 NCCT marker.

Table 3 gives an overview of imaging characteristics stratified according to the evaluated NCCT markers and the frequency of concomitantly present NCCT. Patients with island sign, black hole sign and blend sign had larger ICH volumes than patients without. The distribution of functional outcome evaluated by mRS score at 12 months stratified according to the evaluated NCCT markers (present vs. absent) is shown in Figure 2.

**Table 3:** Characteristics of patients divided according to the presence of NCCT markers.

	Any sign (*n* = 255)	Irregular shape (*n* = 224)	Heterogenous density (*n* = 180)	Hypodensities (*n* = 169)	Island sign (*n* = 62)	Blend sign (*n* = 15)	Black hole sign (*n* = 37)
Age, median (IQR), y	68.0 (59.0–77.0)	68.5 (59.0–76.8)	68.0 (59.0–76.0)	67.0 (58.5–75.5)	67.0 (58.0–75.5)	64.0 (55.0–72.0)	67.0 (60.5–78.5)
Female sex, *n* (%)	101 (39.6%)	91 (40.6%)	65 (36.1%)	59 (34.9%)	19 (30.6%)	6 (40%)	14 (37.8%)
**Timing (h), median (IQR)**							
Symptom onset to admission	3.0 (1.1–6.8)	2.9 (1.1–7.3)	2.7 (1.1–5.4)	3.0 (1.3–6.3)	4.0 (1.3–8.4)	2.9 (1.5–6.4)	3.2 (1.0–6.3)
Symptom onset to diag. CT	3.5 (1.5–7.3)	3.6 (1.5–8.0)	3.2 (1.5–6.1)	3.5 (1.6–6.7)	4.4 (1.7–8.8)	3.4 (1.6–6.7)	3.5 (1.4–6.5)
Diag. CT to follow-up CT	20.7 (13.1–27.2)	20.4 (13.0–26.4)	20.4 (13.0–28.0)	20.4 (13.3–28.1)	23.2 (16.8–30.7)	24 (14.0–29.2)	20.4 (12.7–28.2)
**Imagine**							
1. ICH volume, median (IQR)	16.5 (7.9–31.6)	17.1 (8.7–34.2)	17.8 (9.5–37.2)	19.2 (9.9–39.1)	33.9 (17.5–53.3)	42.0 (17.9–73.8)	38.0 (17.9–57.8)
IVH, *n* (%)	166 (65.1%)	150 (67.0%)	124 (68.9%)	116 (68.6%)	43 (69.4%)	8 (53.3%)	27 (73.0%)
2. ICH volume, median (IQR)	16.0 (7.7–37.2)	17.3 (8.0–42.0)	18.6 (9.8–47.0)	22.7 (10.4–47.8)	37.9 (18.7–64.6)	48.0 (24.6–78.5)	44.2 (17.1–68.0)
HE 33% and/or 6 mL, *n* (%)	41 (16.1%)	37 (16.5%)	40 (22.2%)	36 (21.3%)	14 (22.6%)	3 (20.0%)	9 (24.3%)
**NCCT markers, *n* (%)**							
Irregular shape	224 (87.8%)		155 (86.1%)	142 (84.0%)	58 (93.5%)	15 (100.0%)	31 (83.8%)
Heterogenous density	180 (70.6%)	155 (69.2%)		140 (82.8%)	52 (83.9%)	13 (86.7%)	35 (94.6%)
Hypodensities	169 (66.3%)	142 (63.4%)	140 (77.8%)		49 (79.0%)	14 (93.3%)	36 (97.3%)
Island sign	62 (24.3%)	58 (25.9%)	52 (28.9%)	49 (29.0%)		4 (26.7%)	16 (43.2%)
Blend sign	15 (5.9%)	15 (6.7%)	13 (7.2%)	14 (8.3%)	4 (6.5%)		5 (13.5%)
Black hole sign	37 (14.5%)	31 (13.8%)	35 (19.4%)	36 (21.3%)	16 (25.8%)	5 (33.3%)	

Baseline characteristics of all patients divided according to the presence of different NCCT (non-contrast CT) markers. *n*, number of patients; SD, standard deviation; IQR, Interquartile range; CI, confidence interval; SMD, standardized mean differences; ICH, intracerebral hemorrhage, IVH, intraventricular hemorrhage; HE, Hematoma enlargement.

### HE and NCCT markers

Table 4 shows the baseline characteristics of patients with or without HE (cutoff 6 mL and/or 33%). Significant associations with HE were present for prior use of OAC [34.8% (16/46) vs. 10.5% (29/276); AD: 24.3; 95%CI (10.0 to 38.5); SMD: 0.60], with earlier admission after symptom onset [1.7 (0.9–4.1) h vs. 3.5 (1.4–8.7) h; AD: −1.8, 95%CI (−3.5 to −0.04); SMD: −0.40] and earlier diagnostic CT [2.2 (1.2–5.1) h vs. 4.1 (1.7–9.2) h; AD: −1.9, 95%CI (−3.6 to −0.2); SMD: −0.39]. There was no difference in the time between diagnostic CT and follow-up CT [18.2 (9.6–26.5) h vs. 21.7 (14.6–29.3) h; AD: −3.3, 95%CI (−6.9 to 0.2); SMD: −0.17]. Patients with HE had larger ICH volumes at diagnostic CT [17.8 (10.8–37.6) vs. 10.2 (4.9–25.4); AD: 7.6, 95%CI (1.8 to 13.5); SMD: 0.27] and on follow-up CT [34.6 (16.3–62.0) vs. 9.8 (4.6–25.8); AD: 24.9, 95%CI (18.3 to 31.5); SMD: 0.90]. Two NCCT markers were significantly associated with HE, i.e., heterogenous density [87.0% (40/46) vs. 50.7% (140/276); AD: 36.2, 95%CI (24.9 to 47.6); SMD: 0.85] and hypodensities [78.3% (36/46) vs. 48.2 (133/276); AD: 30.1, 95%CI (16.8 to 43.4); SMD: 0.65].

**Table 4:** Comparison of patients without versus patients with HE.

	HE absent (*n* = 276)	HE present (*n* = 46)	Absolute difference(95%CI)	SMD
Age, median (IQR), y	69.0 (60.0–77.0)	68.0 (57.8–77.0)	−2.0 (−7.8 to 3.8)	−0.08
pre-mRS, median (IQR)	1 (0–2)	1 (0–1)	0.0 (−0.6 to 0.6)	−0.06
Female sex, *n* (%)	114 (41.3%)	15 (32.6%)	−8.7 (−23.4 to 6.0)	−0.18
**Medical history, *n* (%)**				
Arterial hypertension	253 (91.7%)	44 (95.7%)	4.0 (−2.7 to 10.7)	0.16
Heart failure	39 (14.1%)	8 (17.4%)	3.3 (−8.4 to 15.0)	0.09
Prior ischemic stroke/TIA	57 (20.7%)	10 (21.7%)	1.1 (−11.7 to 14.0)	0.03
Prior Bleeding complications	18 (6.5%)	4 (8.7%)	2.1 (−6.5 to 10.8)	0.08
Hepatic insufficiency	26 (9.4%)	5 (10.9%)	1.4 (−8.2 to 11.1)	0.05
Renal failure	42 (15.2%)	10 (21.7%)	6.5 (−6.1 to 19.2)	0.17
Diabetes mellitus	84 (30.4%)	10 (21.7%)	−8.7 (−21.8 to 4.4)	−0.20
APT	85 (30.8%)	10 (21.7%)	−9.1 (−22.2 to 4.0)	−0.21
OAC	29 (10.5%)	16 (34.8%)	24.3 (10.0 to 38.5)	0.60
**Status at hospital admission**				
GCS, median (IQR)	12 (7–15)	11 (5–13)	−1.0 (−3.1 to 1.1)	−0.19
NIHSS, median (IQR)	14 (8–24)	18 (13–28)	4.0 (0.0 to 8.0)	0.28
Syst. blood pressure, median (IQR)	170 (150–190)	170 (154.3–199.3)	0.0 (−12.9 to 12.9)	0.10
Dia. blood pressure, median (IQR)	91.5 (80.0–105.5)	99.0 (78.0–114.5)	6 (−1.5 to 13.5)	0.18
Mean blood pressure, mean (SD)	118.6 (21.9)	121.4 (26.0)	2.0 (−7.7 to 11.7)	0.12
maxICH score, median (IQR)	4 (3–5)	4.5 (4–5)	1 (0.0 to 2.0)	0.49
**Timing hours, median (IQR)**				
Symptom onset to admission	3.5 (1.4–8.7)	1.7 (0.9–4.1)	−1.8 (−3.5 to −0.04)	−0.40
Symptom onset to diag. CT	4.1 (1.7–9.2)	2.2 (1.2–5.1)	−1.9 (−3.6 to −0.2)	−0.39
Diag. CT to follow-up CT	21.7 (14.6–29.3)	18.2 (9.6–26.5)	−3.3 (−6.9 to 0.2)	−0.17
**Imagine**				
1. ICH volume (mL), median (IQR)	10.2 (4.9–25.4)	17.8 (10.8–37.6)	7.6 (1.8 to 13.5)	0.27
IVH, *n* (%)	164 (59.4%)	34 (73.9%)	14.5 (0.5 to 28.4)	0.31
2. ICH volume (mL), median (IQR)	9.8 (4.6–25.8)	34.6 (16.3–62.0)	24.9 (18.3 to 31.5)	0.90
**NCCT markers, *n* (%)**				
Any sign	214 (77.5%)	41 (89.1%)	11.6 (1.3 to 21.8)	0.31
Irregular shape	187 (67.8%)	37 (80.4%)	12.7 (0.0 to 25.4)	0.29
Heterogenous density	140 (50.7%)	40 (87.0%)	36.2 (24.9 to 47.6)	0.85
Hypodensities	133 (48.2)	36 (78.3%)	30.1 (16.8 to 43.4)	0.65
Island sign	48 (17.4%)	14 (30.4%)	13.0 (−1.0 to 27.1)	0.31
Blend sign	12 (4.3%)	3 (6.5%)	2.2 (−5.4 to 9.7)	0.10
Black hole sign	28 (10.1%)	9 (19.6%)	9.4 (−2.6 to 21.4)	0.27

Comparison of patients with HE (hematoma enlargement) vs. without HE (cutoff 33% and/or 6 mL). Absolute differences are provided in percent for frequency data and for scales or continuous variables as mean differences of the according measurement unit. Standardized mean differences (SMD) are given to compare the difference between patients with and without HE. *n*, number of patients; SD, standard deviation; IQR, Interquartile range; CI, confidence interval; SMD, standardized mean differences; ICH, intracerebral hemorrhage; IVH, intraventricular hemorrhage; APT, prior antiplatelet drug use; OAC, prior oral anticoagulation drug use; premRS, modified Ranking scale sore before stroke; GCS, Glasgow Coma Scale (ranging from 3, comatose, to 15, alert); NIHSS, National Institutes of Health Stroke Scale (ranging from 0, no stroke symptoms, to 42, severe stroke); syst. Blood pressure, systolic blood pressure; dia. Blood pressure, diastolic blood pressure, mean blood pressure, mean arterial blood pressure; max-ICH Score; maximally treated ICH Score; NCCT, non-contrast CT; Any sign, the presence of at least 1 NCCT marker.

### Independent predictors of unfavorable outcome and HE

**Figure 3:**
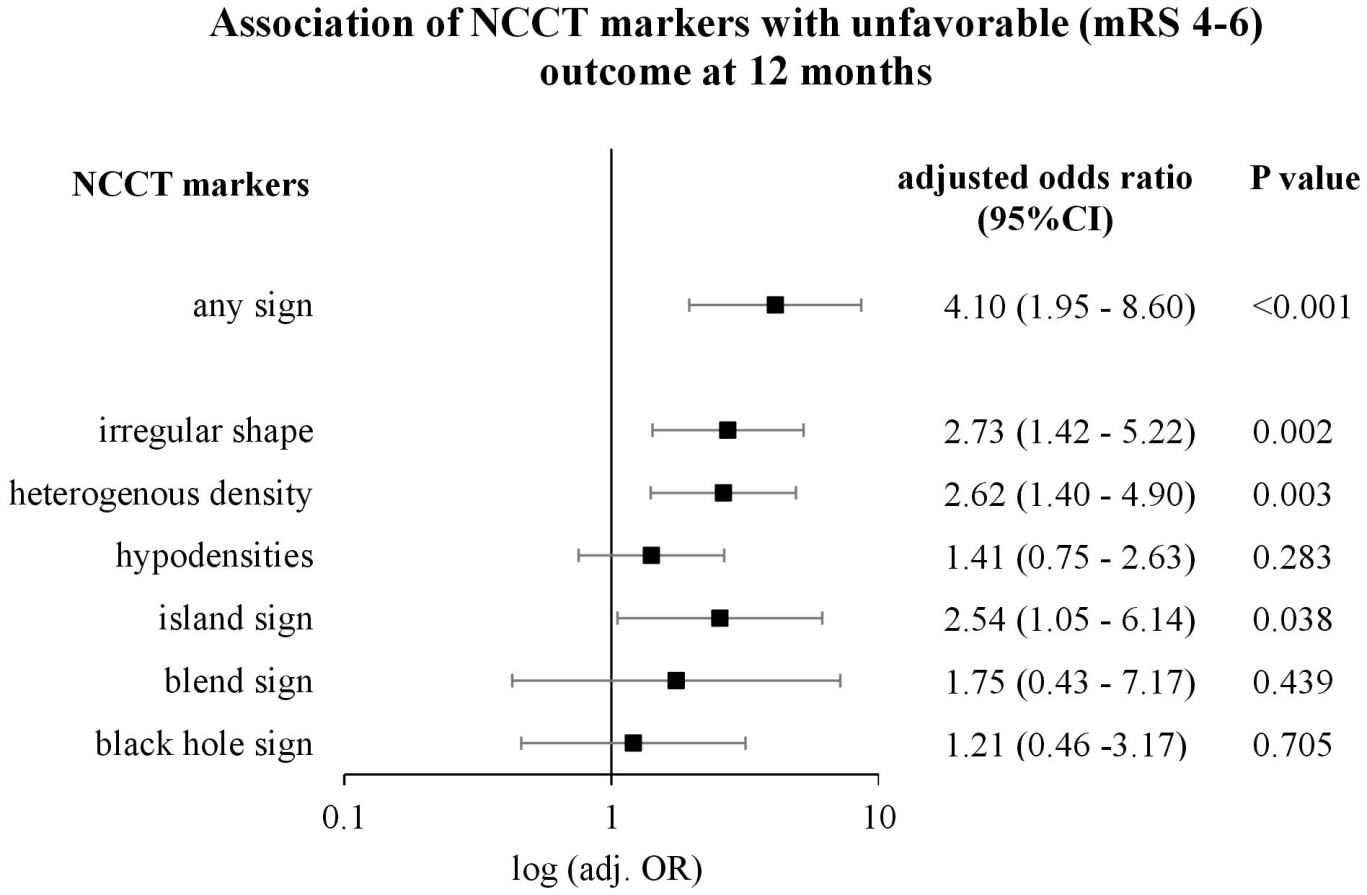
Association of NCCT markers with unfavorable outcome (mRS 4–6) at 12 months. Logarithmic forest plot, which shows adjusted odds ratios (OR), confidence interval (CI), and *p*-values of non-contrast CT (NCCT) markers for long-term outcome after adjusting for age, baseline intracerebral hemorrhage volume (ICH), hematoma enlargement (HE) (defined as a 33% and/or 6 mL increase), National Institutes of Health Stroke Scale (NIHSS) ranging from 0, no stroke symptoms, to 42, severe stroke, pre-stroke modified Ranking Scale (pre-mRS) and intraventricular hemorrhage (IVH).

Upon multivariable modeling, we identified independent associations with unfavorable functional outcome for age (aOR: 5.29, 95%CI: 2.79–10.02; *p* < 0.001), pre-mRS score (aOR: 3.84, 95%CI: 1.89–7.81; *p* < 0.001), IVH (aOR: 4.29, 95%CI: 2.39–7.70; *p* < 0.001) and baseline ICH volume (aOR: 4.25, 95%CI: 2.21–8.18; *p* < 0.001). In the second step, we conducted an adjusted analysis for each NCCT marker with unfavorable outcome separately, which showed significant associations for irregular shape (aOR: 2.73, 95%CI: 1.42–5.22; *p* = 0.002), heterogenous density (aOR: 2.62, 95%CI: 1.40–4.90; *p* = 0.003) and island sign (aOR: 2.54, 95%CI: 1.05–6.14; *p* = 0.038), as shown in Figure 3. The absence of any NCCT marker was associated with significantly reduced odds of achieving unfavorable outcome at 12 months (aOR: 0.24, 95%CI: 0.12–0.51; *p* < 0.001).

**Figure 4:**
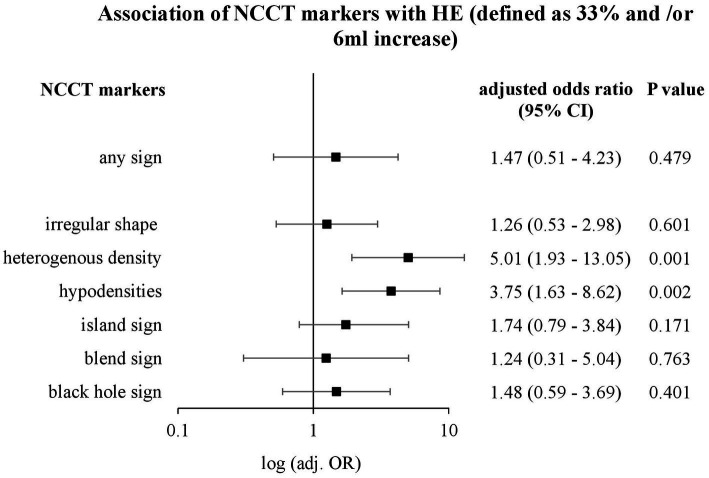
Association of NCCT markers with HE (defined as 33% and/or 6 mL increase). Logarithmic forest plot, which shows odds ratio (OR), confidence interval (CI), and *p*-value of non-contrast CT (NCCT) markers for hematoma enlargement (HE) after adjusting for ICH baseline volume, prior use of antiplatelet drugs, prior use of anticoagulant drugs, systolic blood pressure, time from symptom onset to diagnostic CT, and time from diagnostic to follow-up CT.

Upon multivariable modeling for independent associations with HE, baseline ICH volume (aOR: 2.6, 95%CI: 1.29–5.24; *p* = 0.007), prior use of OAC (aOR: 4.83, 95%CI: 2.23–10.43; *p* < 0.001) and time from symptom onset to diagnostic CT (aOR: 0.51, 95%CI: 0.25–1.01; *p* = 0.05) were significantly associated with HE. Upon adjusted analyses for each NCCT marker with HE, significant associations were present for heterogenous density (aOR: 5.01, 95%CI: 1.93–13.05; *p* = 0.001) and hypodensities (aOR: 3.75, 95%CI: 1.63–8.62; *p* = 0.002), as shown in Figure 4.

## Discussion

The present study, for the first time, evaluated the association of NCCT markers with long-term functional outcome solely in deep ICH patients. This important subgroup of ICH patients is characterized by the highest risk for HE (8), more rapid HE-dynamics (13), smaller volume increases leading to poorer functional outcome (8), and a greater societal burden due to younger age (8). We identified the NCCT marker heterogenous density as clinically meaningful in deep ICH patients, increasing the odds for unfavorable functional long-term outcome by 2.5-fold and for HE by 5-fold.

There are several well-established predictors of functional outcome, including age, neurological assessment, hematoma volume and hematoma expansion (5, 29–32). Yet, the very first step for ICH diagnosis in most patients is a non-contrast CT scan. Hence, the availability of easy-to-use imaging biomarkers that are robustly associated with clinical outcome reflects a very important step in hyperacute care. Furthermore, if this surrogate marker is also specific for the most important treatment target HE, then this parameter could serve as an important and clinically meaningful predictor both ways. According to a recent meta-analysis, NCCT markers such as irregular shape and density, black hole sign, blend sign, hypodensities, and island sign were all associated with poor short-term outcome and an increased risk for HE, which, however, was based on unadjusted pooled estimates (15). In our study, after robust adjustments, only the density markers (heterogenous density) and shape markers (irregular shape and island sign) were independently associated with poor long-term outcome. At the same time, only heterogenous density and hypodensities were independently associated with HE. These findings suggest a simplification to only one density marker, that is heterogenous density, which serves as the only parameter associated with both outcomes and, therefore, may represent the ideal radiologic biomarker in patients with deep ICH. Heterogenous density of ICH is conventionally assessed on a visual analog scale (21) and hence may be easily integrated into clinical practice or even into automated analyses using artificial intelligence-based methodologies. Furthermore, it remains to be determined to what extent aggressive treatment interventions, such as systolic blood pressure lowering or hemostatic approaches, may translate to larger effect sizes in patients with present NCCT markers to prevent HE.

The complexity of the increasing number and partially overlapping definitions of NCCT markers evaluated in recent years, as well as different standards of HE, may have hindered clinical applicability, especially translation into clinical trials of hyper-acute management as important selection criteria. Importantly, recent data suggest that time course (e.g., symptom onset to CT) affects the sensitivity and specificity of different NCCT markers. In line with a recent study, which showed that hypodensity and heterogenous density makers had a higher sensitivity within a 2-h time window, we report that these subgroups of patients (heterogenous density and hypodensity positive) arrived at the hospital the earliest, at a median of 2.7 and 3h, respectively (33). In theory, this would lead to two clinically meaningful aspects: (1) the ability to identify high-risk patients without known symptom onset or after wake-up constellations in ICH and (2) the identification of patients with a greater potential that aggressive interventions exert larger effect sizes on HE prevention and functional long-term outcome. Increasingly, researchers suggest a combination of markers and time measures as ideal for predicting HE, and therefore, our findings would suggest that in deep ICH, the NCCT markers, heterogenous density and hypodensities, would be valuable to predict HE (34).

For our research, we used granular data, robust and standardized methodology and validated definitions for imaging analyses; specifically, we applied the most commonly used definitions of hematoma expansion after volumetric analyses (17, 20). For NCCT markers, we strictly followed the Standards for Detecting, Interpreting, and Reporting Noncontrast Computed Tomographic Markers of Intracerebral Hemorrhage Expansion by Morotti et al. (17). Additionally, we used prospectively collected data on long-term functional outcome, which may provide increased generalizability of our findings. Nevertheless, our research has several limitations. First, due to the retrospective design, the time points of follow-up imaging were not standardized. Second, we did not execute in-depth analyses of the IVH extent, which deserves future investigations (35, 36). Third, accompanying CTA imaging for spot sign evaluation was not available in all cases; hence, a comparison of diagnostic performance to NCCT markers was not feasible. Finally, since the examined population consisted mainly of white Europeans, it is questionable whether our findings can be transferred to other ethnic groups. Many prior studies largely investigated Asian cohorts in which the incidence of ICH is higher compared to other ethnic groups, and moreover, limited data is available regarding ethnic differences in HE dynamics (15, 37, 38).

## Conclusion

NCCT markers are frequent in deep ICH patients and provide important clinical implications. Specifically, markers defined by diverging intra-hematomal densities provided associations with a 5-times higher risk for HE and a 2.5-times higher likelihood of unfavorable functional long-term outcome. Hence, these markers allow the identification of high-risk patients with deep ICH for clinical deterioration due to HE.

## Data availability statement

The datasets presented in this article will be made available upon reasonable request and in adherence with institutional data sharing regulations. Requests to access the datasets should be directed to joji.kuramatsu@uk-erlangen.de.

## Ethics statement

The studies involving humans were reviewed and approved by the Ethics committee of the Friedrich-Alexander-University Erlangen-Nuremberg, approval number: 115_17B. The studies were conducted in accordance with the local legislation and institutional requirements. The participants provided their written informed consent to participate in this study.

## Author contributions

KK: Conceptualization, Data curation, Formal analysis, Investigation, Writing – original draft. SH: Conceptualization, Data curation, Formal analysis, Investigation, Writing – original draft. MX: Data curation, Investigation, Writing – review & editing. JS: Data curation, Writing – review & editing, Methodology, Project administration. AM: Data curation, Writing – review & editing, Project administration, Validation. SB: Data curation, Writing – review & editing, Validation. SL: Data curation, Investigation, Writing – review & editing, Project administration. BV: Data curation, Investigation, Methodology, Writing – review & editing, Project administration. TE: Conceptualization, Data curation, Writing – review & editing, Methodology, Supervision, Validation. BK: Data curation, Writing – review & editing, Project administration, Supervision. JK: Conceptualization, Formal analysis, Supervision, Writing – original draft, Investigation, Methodology, Project administration, Validation.

## Footnotes

^1^ http://www.stata.com
