# Supplementary material for: Association of non-contrast CT markers with long-term functional outcome in deep intracerebral hemorrhage
Source: Front Neurol. 2024 Jan 11;14:1268839. doi: 10.3389/fneur.2023.1268839 (PMC10810138; doi:10.3389/fneur.2023.1268839)
Supplement: Supplementary file 1 [file Table_1.DOCX]

**Supplemental Table 1: Comparison of included vs excluded patients**

|  | **Included patients (n=322)** | **Excluded patients (n=265)** | **SMD** |
| --- | --- | --- | --- |
| Age, median (IQR) | 69.0 (59.6-77.0) | 74.0 (65.0-81.0) | 0.31 |
| Female sex, n (%) | 129 (40.1%) | 127 (47.9%) | 0.16 |
| **Medical history, n (%)** |  |  |  |
| Arterial Hypertension | 297 (92.2%) | 220 (83.0%) | 0.32 |
| Heart failure | 47 (14.6%) | 41 (15.5%) | 0.02 |
| Prior Ischemic stroke/TIA | 67 (20.8%) | 63 (23.8%) | 0.07 |
| Prior ICH/Bleeding complications | 22 (6.8%) | 21 (7.9%) | 0.04 |
| Hepatic insufficiency | 31 (9.6%) | 27 (10.2%) | 0.02 |
| Renal failure | 52 (16.1%) | 48 (18.1%) | 0.05 |
| Diabetes Mellitus | 94 (29.2%) | 68 (25.7%) | 0.05 |
| APT | 95 (29.5%) | 65 (24.5%) | 0.09 |
| OAC | 45 (14.0%) | 65 (24.5%) | 0.27 |
| **Status at hospital admission** |  |  |  |
| GCS, median (IQR) | 12 (6-14) | 12 (4-15) | 0.09 |
| NIHSS, median (IQR) | 15 (9-24) | 17 (7-32) | 0.08 |
| maxICH Score, median (IQR) | 4 (3-5) | 5 (3-7) | 0.24 |
| **Imagine** |  |  |  |
| 1. ICH volume (ml), median (IQR) | 11.97 (5.1 -27.0) | 17.3 (4.9 – 53.0) | 0.48 |
| IVH, n (%) | 208 (64.6%) | 163 (61.5%) | 0.01 |

Comparison of all included patients (n=322) vs excluded patients (n=265). Patients were excluded because of missing follow-up imaging (<48h), early care limitations, transferals, hematoma evacuation surgery and primary IVH. Absolute differences are provided in percent for frequency data and for scales or continuous variables as mean differences of the according measurement unit. Standardized mean differences (SMD) are given to compare the difference between included and excluded patients* Abbreviations: n, number of patients; SD, standard deviation; IQR, Interquartile range; SMD, standardized mean differences; ICH, intracerebral hemorrhage, APT, prior antiplatelet drug use; OAC, prior oral anticoagulation drug use; premRS, modified Ranking scale sore before stroke; GCS, Glasgow Coma Scale (ranging from 3, comatose, to 15, alert); NIHSS, National Institutes of Health Stroke Scale (ranging from 0, no stroke symptoms, to 42, severe stroke); max-ICH Score; maximally treated ICH Score; IVH, intraventricular hemorrhage.
